# l-Citrulline Supplementation: Impact on Cardiometabolic Health

**DOI:** 10.3390/nu10070921

**Published:** 2018-07-19

**Authors:** Timothy D. Allerton, David N. Proctor, Jacqueline M. Stephens, Tammy R. Dugas, Guillaume Spielmann, Brian A. Irving

**Affiliations:** 1 Pennington Biomedical Research Center, Baton Rouge, LA 70808, USA; Timothy.Allerton@pbrc.edu (T.D.A.); jsteph1@lsu.edu (J.M.S.); gspielmann@lsu.edu (G.S.); 2 Department of Kinesiology, Pennsylvania State University, University Park, PA 16802, USA; dnp3@psu.edu; 3 Department of Comparative Biomedical Sciences, School of Veterinary Medicine, Louisiana State University, Baton Rouge, LA 70803, USA; tammydugas@lsu.edu; 4 Department of Kinesiology, Louisiana State University, Baton Rouge, LA 70803, USA

**Keywords:** supplements, therapeutics, interventions, watermelon, nitric oxide, arginine, endothelial function, flow mediated dilation, mitochondria, enterocytes, liver, adipocytes, muscle, immune cells, obesity, aging, hypertension, inflammation, insulin resistance, diabetes, cardiovascular disease

## Abstract

Diminished bioavailability of nitric oxide (NO), the gaseous signaling molecule involved in the regulation of numerous vital biological functions, contributes to the development and progression of multiple age- and lifestyle-related diseases. While l-arginine is the precursor for the synthesis of NO by endothelial-nitric oxide synthase (eNOS), oral l-arginine supplementation is largely ineffective at increasing NO synthesis and/or bioavailability for a variety of reasons. l-citrulline, found in high concentrations in watermelon, is a neutral alpha-amino acid formed by enzymes in the mitochondria that also serves as a substrate for recycling l-arginine. Unlike l-arginine, l-citrulline is not quantitatively extracted from the gastrointestinal tract (i.e., enterocytes) or liver and its supplementation is therefore more effective at increasing l-arginine levels and NO synthesis. Supplementation with l-citrulline has shown promise as a blood pressure lowering intervention (both resting and stress-induced) in adults with pre-/hypertension, with pre-clinical (animal) evidence for atherogenic-endothelial protection. Preliminary evidence is also available for l-citrulline-induced benefits to muscle and metabolic health (via vascular and non-vascular pathways) in susceptible/older populations. In this review, we examine the impact of supplementing this important urea cycle intermediate on cardiovascular and metabolic health outcomes and identify future directions for investigating its therapeutic impact on cardiometabolic health.

## 1. Introduction

**Figure 1:**
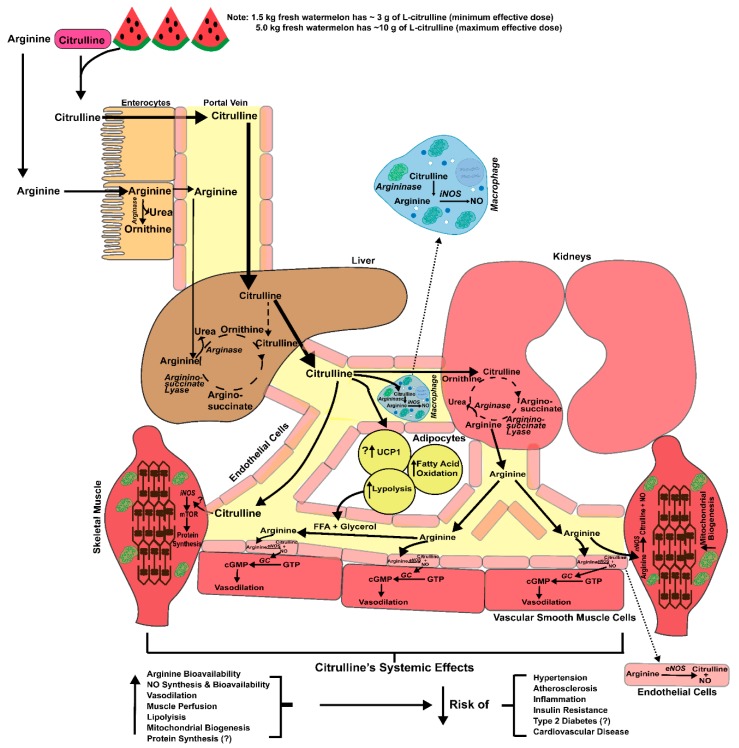
Comparison of oral l-citrulline (via pharmaceutical/nutraceutical grade l-citrulline or watermelon products) versus oral l-arginine. The activity of the arginase enzyme located in the enterocytes of intestines and liver (first-pass extraction) substantially reduces the availability of oral l-arginine, instead yielding increased urea and l-ornithine production. l-citrulline is not acted on by arginase enzyme or first-pass extraction but is converted to l-arginine by argininosuccinate lyase in the kidneys. Increased circulating l-arginine serves a substrate for the eNOS to produce nitric oxide (NO) and increase smooth muscle vasodilation. l-citrulline may directly activate inducible nitric oxide synthase (iNOS) in skeletal muscle and increase protein synthesis via mTOR activation. l-citrulline may indirectly activate neuronal nitric oxide synthase (nNOS) in skeletal muscle leading to increases in NO and stimulation of mitochondrial biogenesis. l-citrulline has reported actions on adipose tissue to increase lipolysis, fatty acid oxidation, and uncoupling protein 1 (UCP1) expression. l-citrulline has also been reported to indirectly activate iNOS in activated macrophages and increase NO production. l-citrulline’s systemic effects positively impact hypertension, atherosclerosis, inflammation, insulin resistance, type 2 diabetes, and cardiovascular disease. Emerging evidence also suggests that l -citrulline itself can positively impact skeletal muscle and adipose tissue to improve metabolic syndrome. This figure was partially modified from Irving and Spielmann (2016) [13].

Diminished bioavailability of nitric oxide (NO), the gaseous signaling molecule involved in the regulation of numerous vital biological functions, contributes to the development of multiple age- and lifestyle-related risk factors and diseases including hypertension, atherosclerosis, insulin resistance, type 2 diabetes (T2D), and cardiovascular disease [1,2,3,4]. In endothelial cells, NO is synthesized from l-arginine (precursor) by endothelial-nitric oxide synthase (eNOS) generating NO and l-citrulline (products) [5,6,7]. In addition to reductions in NO synthesis, elevations in reactive oxygen species (ROS), especially superoxide (O_2_^−^), can reduce the bioavailability of NO through the generation of peroxynitrite (ONOO^−^), which further promotes endothelial dysfunction that is commonly associated with cardiometabolic diseases [6]. Thus, augmenting l-arginine levels in the circulation may represent a potential therapeutic mechanism to increase NO synthesis and bioavailability. However, oral l-arginine supplementation is largely ineffective due to gastrointestinal and hepatic extraction of l-arginine [8] (Figure 1), as well as a dose-dependent presentation of gastrointestinal distress [9]. Alternatively, oral l-citrulline supplementation consistently increases plasma and tissue levels of l-arginine and NO bioavailability [10,11,12].

l-Citrulline is a neutral, non-essential [14] alpha-amino acid that is an important component of the urea cycle in the liver and kidneys [15]. As a non-protein amino acid, l-citrulline is rarely found in food, but is highly concentrated in watermelon [16]. The concentration of l-citrulline in watermelon grown in the United States can range from 1.6 to 3.5 g/kg of fresh watermelon [16,17,18]. As such, consumption of approximately 1–1.5 kg/day (2.2–3.3 lbs/day) of fresh watermelon would be needed to achieve the minimum effective dose of l-citrulline (3 g/day) and 3.3–5.0 kg/day (7.3–16.5 lbs/day) of fresh watermelon would be needed to achieve the maximum effective dose of l-citrulline (10 g/day) [16,17,18]. Given the growing evidence that endothelial dysfunction has its origins in deficient l-arginine-NO metabolism and given the relative ineffectiveness of l-arginine supplementation on NO metabolism, researchers have begun to explore the potential therapeutic benefits of l-citrulline. l-citrulline is typically supplemented using pharmaceutical/nutraceutical grade l-citrulline, l-citrulline conjugated with malate (1:1 ratio), or as watermelon extract. l-citrulline, as with many other NO-boosting supplements, has received much interest for its potential cardiovascular and anti-hypertensive capabilities [19]. Although recent reviews have eloquently reviewed the impact of citrulline supplementation in health and disease [14,20,21], the present review focuses on l-citrulline’s NO-dependent and NO-independent effects on cardiometabolic outcomes. Moreover, we specifically summarize current literature regarding the benefits of both pharmaceutical/nutraceutical grade l-citrulline and watermelon supplements (juice, water, extract, etc.) on vascular and metabolic physiology and their potential therapeutic impact on cardiometabolic health. We also discuss recent studies that have begun to examine l-citrulline’s direct and indirect effects on clinically relevant aspects of skeletal muscle and adipose tissue metabolism, which are key mediators for the development of cardiometabolic disorders.

## 2. Health Applications

The health-related applications of l-citrulline supplementation are largely predicated on the capacity for l-citrulline to increase l-arginine availability for NO production. NO released from the endothelium as a gas initiates a signaling cascade involving the activation of soluble guanylate cyclase (sGC) to increase cyclic guanosine monophosphate (cGMP) synthesis [22] (Figure 1). Increased levels of cGMP acts as a second messenger to, in the case of NO, increase vasodilation by relaxing the smooth muscle cells of the conduit and resistance arteries [22]. Reduced eNOS expression and NO production/bioavailability has been reported in patients with essential hypertension, healthy older individuals, and heart failure patients [2,23,24]. Moreover, reduced NO bioavailability has both direct and indirect effects on skeletal muscle metabolism that likely contribute to the development of insulin resistance and type 2 diabetes as well as age-related muscle wasting [25,26].

## 3. Pharmacokinetics, Transport and Metabolism

Historically, researchers have considered l-glutamine within enterocytes as the major precursor for the synthesis of l-citrulline and subsequent release into systemic circulation [27]. However, recent data suggest that l-glutamine’s contribution to l-citrulline biosynthesis may be overestimated [28]. On the other hand, there is evidence that enterocytes take up orally ingested l-citrulline and effectively transport the neutral amino acid through the gastrointestinal tract to the portal circulation, likely using the Na^+^-dependent, neutral amino acid, including the ASC or B^0,+^-amino acid transporters located in the enterocytes within the jejunum and ileum [15,29,30]. Quantitatively, l-citrulline is not extracted by the gastrointestinal tract or liver (net uptake~0) [31], which likely facilitates greater down-stream production of NO through the recycling of l-citrulline to the NO precursor l-arginine (Figure 1). In contrast, orally ingested l-arginine is subject to moderate-to-high rates of first-pass extraction both in intestine and liver, likely due to their high expression of arginase [32,33,34], increasing arginine catabolism and limiting systemic increases in circulating levels following its ingestion [8].

To date only a few studies have systematically investigated the pharmacokinetics of l-citrulline supplementation [11,12]. Following oral l-citrulline ingestion, circulating l-arginine concentrations peak after ~1–2 h [11,12]. As has been shown for both l-arginine and l-ornithine, circulating concentrations of l-citrulline return to baseline within 8 h [11]). The higher activity and bioavailability of l-citrulline, compared to l-arginine, is due to several factors. For example, 0.75 grams of l-citrulline ingested twice daily (1.5 g total) increased the l-arginine area under the curve to a similar degree as ingesting 1.6 g of l-arginine twice daily (3.2 g total) (271 vs. 289 μmol·h·L^−1^) [12]. Orally ingested l-citrulline is absorbed by the enterocytes of the small intestine. However, the lack of gastrointestinal distress from l-citrulline compared to l-arginine supplementation may suggest that l-citrulline uptake utilizes a differing transport system. l-arginine is mainly transported across the intestinal membrane through Na+-independent cationic amino acid transporters (CAT-1, 2 and 3) [35]. l-citrulline transport has been demonstrated in enterocytes, macrophages, glial cells, and aortic smooth muscle, with the highest K_m_ (4.1 ± 0.9 mM) reported in the enterocytes [19,30,36]. While a specific l-citrulline transporter has not been identified, the B^0^-transporters have been suggested to play a role in the Na^+^ dependent transport of l-citrulline across the enterocytes [30].

Clinical dose ranging and tolerability studies have also been conducted for l-citrulline supplementation. One such human study demonstrated a tolerance of up to 15 g l-citrulline per day in healthy volunteers [11]. By comparison, high-doses of l-arginine (~13 g) can induce significant gastrointestinal complications [9,21]. However, at 15 g doses of l-citrulline, a lower fractional absorption rate and plasma retention of l-citrulline was observed, potentially due to saturation of its transporters (e.g., ASC or B^0,+^-amino acid transporters) or reduced renal conversion of l-citrulline to l-arginine. As such, the authors suggested a dose of 10 g l-citrulline for clinical use [11]. However, for increasing circulating l-arginine concentrations, doses of l-citrulline as low as 3 g have been shown to be effective [12]. Thus, the minimum effective dose is ~3 g/day, whereas the maximal effective dose may be as high as 10 g/day.

The metabolism of orally ingested l-citrulline is mainly confined to the biosynthesis of l-arginine. Researchers previously thought that most dietary l-citrulline was synthesized via the consumption of l-glutamine through a transamination reaction in the enterocyte of the gastrointestinal tract [37]. However, a recent study using a labeled 2,3,3,4,4 [^2^H_5_] glutamine tracer provided evidence that the contribution of l-glutamine to l-citrulline may be rather modest [28]. Since l-citrulline metabolism in the liver is somewhat compartmentalized to the urea cycle, exogenous l-citrulline typically bypasses hepatic metabolism. Circulating l-citrulline, released from the gut, is absorbed by the proximal tubular cells of the kidney [38]. l-Citrulline is rapidly acted upon by cytosolic arginosuccinate synthase and converted into arginosuccinate which is then converted into l-arginine by arginosuccinate lyase (see Figure 1). This partial urea cycle meets the demand of the body’s l-arginine requirement. De novo synthesis of l-arginine from l-citrulline is essential for downregulating urea formation in the liver during periods of low protein intake to increase nitrogen retention [39].

**Figure 2:**
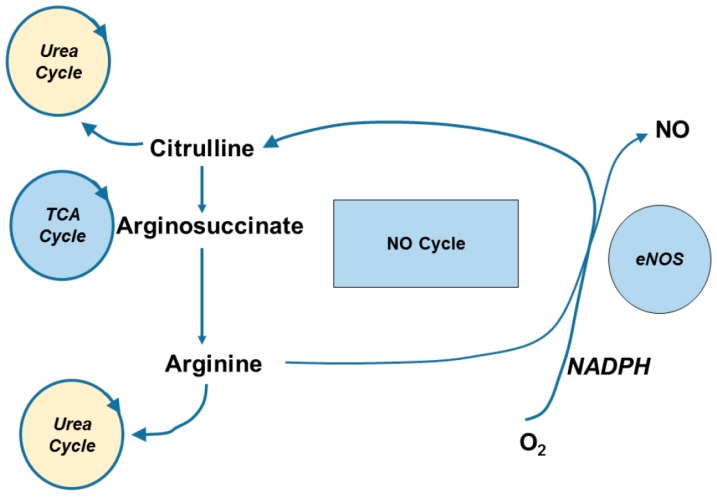
Nitric Oxide (NO) Cycle. Schematic representation of the NO cycle.

Another potential source of l-citrulline is its synthesis via the NO cycle (Figure 2). l-arginine is required for NO formation via eNOS, iNOS, and nNOS. Hydrolysis of the intermediate nitrosoarginine from l-arginine yields NO and l-citrulline [15]. This is particularly useful in endothelial cells via eNOS [40,41] and activated macrophages via iNOS [42] to sustain l-citrulline as a precursor to l-arginine to produce NO [36] (Figure 1). However, the recycling of l-citrulline to l-arginine does not appear as viable in cell types that have suboptimal uptake of l-citrulline from plasma, such as aortic smooth muscle cells. Indeed, aortic smooth muscle cells take up l-citrulline at a relatively slow rate compared to l-arginine, due in part to its transport through a low affinity (K_m_ 1.6 mM) transporter [36]. Consistent with this notion, Hattori and colleagues reported that physiologically high concentrations of l-citrulline are necessary to maximally stimulate iNOS activity in cultured smooth muscle cells [43]. Under inflammatory conditions (Lipopolysaccharides and Interferon-γ stimulation), l-arginine itself is capable of inducing NOS via its increased transport.

## 4. Vasoprotective Effects of l-Citrulline

### 4.1. Endothelial Vasodilator Function

l-citrulline increases NO biosynthesis indirectly by increasing l-arginine synthesis, which in turn may lead to improved endothelial vasodilator function [12,24,44]. The reduction in synthesis of eNOS is thought to play a major role in the endothelial dysfunction associated with aging, menopause, and cardiometabolic disease [1,45,46]. Work in rodent models of reduced l-arginine bioavailability has demonstrated that l-citrulline, but not l-arginine supplementation, increased NO synthesis and microcirculatory (gut villi) blood flow [47]. Furthermore, in a rodent model of spontaneous hypertension and chemical disruption of NO bioavailability, l-citrulline restored levels of NO by increases l-arginine/asymmetrical dymethylarginine (ADMA) ratio [48,49,50]. These pre-clinical studies support the hypothesis that endothelial function may be enhanced by the capability of l-citrulline supplementation to increase l-arginine levels.

In healthy, young participants increased levels of l-arginine, nitrate/nitrite, and cGMP activities have been consistently demonstrated after l-citrulline supplementation [11,12,51]. However, improvement in endothelial function, as measured by brachial artery flow mediated dilation (FMD), have not been reported with acute or short-term (~7 days) administration of l-citrulline, despite significantly increased l-arginine bioavailability and increased urinary nitrate/nitrite (NOX) [12,24,44]. One study that provided young, healthy subjects with 10 g of l-citrulline (i.e., the maximally clinically effective dose) demonstrated increased de novo l-arginine and NO synthesis but did not observe increased forearm blood flow during reactive hyperemia measured by plethysmography [24]. Both the acute time course of these studies and the healthy state of the participants may explain the lack of benefit of l-citrulline supplementation under these conditions.

Age-related endothelial dysfunction is associated with a reduced bioavailability of l-arginine and reduction in eNOS synthesis [1,41,46]. Older adults with heart failure increased de novo l-arginine and NO synthesis after acute ingestion of l-citrulline (10 g), but these synthesis rates were still low when compared to young subjects [24]. When compared to the placebo conditions, l-citrulline supplementation also did not improve forearm blood flow during reactive hyperemia measured by plethysmography in these subjects. Likewise, another study in elderly male subjects measured blood flow at rest and post-exercise by providing l-citrulline combined with whey protein, compared to whey protein alone or whey protein with additional non-essential amino acids [44]. No increases in plasma nitrate/nitrite or limb blood flow were observed during resting or exercise postprandial periods [31]. As is the case with young healthy participants, the length of treatment may be a mediating factor in determining l-citrulline’s lack of efficacy in improving FMD. As an example, in a separate study, l-citrulline supplementation at 800 mg/day for 8 weeks was necessary for elevating plasma l-arginine levels and improving FMD [52]. However, in patients with vasospastic angina, 8 weeks of l-citrulline (800 mg/day) was sufficient to improve l-arginine/ADMA levels and FMD [52]. The variability in changes in FMD in response to l-citrulline supplementation reflect similar investigations into l-arginine supplementation [53]. There are several possible explanations for the lack of consistent observations in this field of study. Both animal and human studies demonstrate a significant relationship between improved FMD and increase l-arginine/ADMA ratio. There is also the possibility that age-associated endothelial dysfunction is not mediated by reduced NO production due to high ADMA levels and therefore, l-citrulline or l-arginine supplementation may not be expected to improve endothelial function under these conditions [54].

### 4.2. Protection against Endothelial Damage

Endothelial dysfunction, often associated with obesity-induced insulin resistance, is thought to be a major factor in the development of cardiovascular disease [45]. Dietary factors, such as high fat/high cholesterol diet, adipocyte derived factors, and aging have also been implication in the promotion of low-grade inflammation and further exacerbated endothelial dysfunction, contributing to the development of cardiovascular disease [45,55,56,57,58]. Studies in rats fed a high fat/high cholesterol diet observed an elevation of the liver enzymes aspartate aminotransferase (AST) and alanine aminotransferase (ALT), indicative of liver stress/damage [59]. Subsequent oral l-citrulline or l-arginine treatment increased high density lipoprotein (HDL) levels, reduced serum AST/ALT, and confirmed some modest but favorable, structural changes in the endothelial structure of the thoracic aorta [59]. These data support the notion that l-citrulline may protect against the liver damage and endothelial dysfunction induced by chronic exposure to a high fat/high cholesterol diet.

Reversal of deficient l-arginine levels via l-citrulline supplementation has been investigated as a therapeutic strategy for preserving endothelial function in rodents consuming an atherogenic diet. Both wild type and apoE^−^/^−^ mice fed a high fat/high cholesterol diet exhibited increased plasma and tissue arginase activity, leading to a reduction in l-arginine bioavailability [60]. In rats fed a Western diet (high fat/high fructose), enrichment with l-citrulline (1 g/kg) reduced hepatic microvesicular lipid droplets and circulating triacylglycerol [61]. In vitro studies in porcine coronary artery endothelium demonstrated that l-citrulline (100 μmol/L) treatment preserved eNOS production following ADMA treatment [62]. l-citrulline preserves endothelial function in rabbits during exposure to a high cholesterol diet by decreasing the production of superoxide and associated oxygen-sensitive proteins ELK-1 and p-CREB [63]. Collectively, these studies in diverse animal models have demonstrated the critical role of NO in endothelial protection against atherogenic dietary conditions and the viable role of l-citrulline in promoting increased endogenous NO production, as well as in reducing the deleterious impact of oxidative stress on NO bioavailability.

### 4.3. Antioxidant and Anti-Inflammatory Effects

In vascular tissues the generation of ROS is driven by nicotinamide adenine dinucleotide phosphate oxidases, which promote platelet aggregation, reduce endothelium-mediated relaxation, and induce pathological vascular remodeling [64]. The mechanisms of l-citrulline to improve endothelial dysfunction, in conditions such as atherosclerosis, are most likely mediated via direct reduction of hydroxyl radical formation, direct action on vascular smooth muscle, and indirect action of NO synthesis. The antioxidant effects of l-citrulline can be viewed in two different ways: NO-dependent and NO-independent pathways. As previously mentioned, l-citrulline is capable of increasing eNOS in endothelial cells, which in turn reduces ROS formation [65]. Nonetheless, l-citrulline possess qualities, as an amino acid, which make it a functional antioxidant under certain physiological conditions.

According to the Haber-Weiss and Fenton reactions, the production of superoxide is coupled with the formation of hydroxyl radicals (HO^−^) [66]. Hydroxyl radical formation is highly upregulated during ischemia/low oxygen availability in tissues supplied by atherosclerotic blood vessels [62]. l-citrulline can reduce hydroxyl radical formation, independent of NO, by directly interacting with hydroxyl radicals via the alpha-amino acids in its protonated NH_3_ state leading to water formation [67,68]. Interestingly, l-citrulline, under ex vivo conditions, performs as a more effective scavenger of ROS mediated endothelial dysfunction at concentrations between 100 μM and 3 mM [68]. However, the effects of l-citrulline on endothelial protection at higher concentrations (30 mM) were absent.

The source of the oxidative stress (superoxide vs. hydroxyl radicals) will likely determine the effectiveness of l-citrulline to attenuate endothelial damage and dysfunction. Therefore, the antioxidant capacity of l-citrulline, and l-arginine, are dependent on the nature of the oxidative stress. The therapeutic actions of l-citrulline are likely mediated by its relative concentration in vivo, especially considering evidence that l-citrulline at higher concentration did not impart protective effects.

l-citrulline supplementation is also likely to provide indirect benefits to vascular health by modulating chronic low-grade inflammation. Indeed, oral l-citrulline ingestion has been shown to reduce serum inflammatory cytokine concentrations, such as IL-6, tumor necrosis factor (TNF)-alpha, and C-reactive protein in both aged animals [69] and humans [70]. Although the exact mechanisms underlying the citrulline-mediated improvements in systemic inflammation remain unknown, Breuilard et al. (2015) recently advocated that serum l-citrulline may exert its health benefits by dampening macrophage cytokine production [71]. This elegant study showed that peritoneal macrophages isolated from Zucker Diabetic Fatty (ZDF) rats secreted significantly less TNF-alpha when stimulated with increasing concentrations of l-citrulline in vitro [71]. This is further supported by in vivo models of sepsis where l-citrulline supplementation induces selective reductions in pro-inflammatory cytokines (IL-6) but preserves anti-inflammatory cytokine (IL-10) production [72] and NO production [73].

In addition to its impact on innate immunity, l-citrulline supplementation appears to regulate adaptive immunity and further reduce cytokine-induced low-grade inflammation. Emerging research on immune energetics has highlighted the importance of metabolic regulation on adaptive immune function [74], and recent studies have placed l-citrulline at the center of CD4+ T-cell metabolism, function, and survival [75]. Considering the equal importance of l-arginine in CD8+ T-cell metabolic profile in vivo [76], it can be hypothesized that l-citrulline supplementation will directly and indirectly modulate the metabolism of innate and adaptive immune cells, improve the efficiency of immune responses to pathogen and reduce both the magnitude and duration of inflammatory responses in response to antigenic challenges. Taken together, these studies suggest that l-citrulline supplementation is likely to have great clinical benefits, especially in the context of inflamm-aging, the chronic low-grade inflammation associated with aging [77], which is a well-known aggravating factor for cardiometabolic disorders [78], poor vascular health [79], and overall morbidity and mortality [80].

### 4.4. Effects on Basal and Hyperemic Limb Blood Flow

Although l-citrulline is an effective inducer of l-arginine, NO, and cGMP levels, there are conflicting reports regarding its effectiveness on tissue perfusion during rest or exercise in healthy volunteers [11,12,24,81]. In a study of healthy young men, no differences between an acute combined nitrate-citrulline supplement and placebo were observed in post-ischemia vascular responses, as measured by near-infrared spectroscopy (NIRS) [82]. Other studies in healthy young participants have provided evidence of increased muscle blood flow during moderate intensity exercise after short-term (7 days) l-citrulline supplementation [81,83,84]. It has been speculated that the lack of significant improvement in blood flow and proxy measurements of endothelial function (reactive hyperemic forearm blood flow) in healthy volunteers is likely due to the physiological limits of vessel compliance [24]. Moreover, during (forearm) exercise sympathetic activation increases NO production in an eNOS dependent fashion in vascular endothelial cells, locally overriding systemic vasoconstriction within the arterioles feeding the exercising muscles [85]. This auto-regulatory mechanism is likely intact in healthy volunteers, and thus does not benefit from l-citrulline supplementation.

## 5. Anti-Hypertensive Effects of l-Citrulline

### 5.1. Resting Blood Pressure and Arterial Stiffness

l-Citrulline has been investigated as a potential therapeutic agent to reduce resting blood pressure by increasing endogenous synthesis of l-arginine and ultimately, NO levels. In spontaneously hypertensive rats (SHR), l-citrulline treatment prevent hypertension by increasing the l-arginine/ADMA ratio [48]. Rats and their offspring treated with a selective NOS inhibitor, N^G^-nitro- l-arginine-methyl ester (L-NAME), develop hypertension. L-NAME treatment reduces the bioavailability of NO by first direct inhibition of the NOS enzyme, thus decreasing NO level and lowering l-Arginine/ADMA ratio in the kidney [49]. L-NAME treated rats, and their offspring, eventually develop hypertension. l-citrulline supplementation protect against hypertension in the offspring of L-NAME treated dams by increasing the l-arginine/ADMA ratio in the kidney [50]. These pre-clinical studies suggest that l-citrulline increases renal NO levels, contributing to the prevention of hypertension.

Both pharmaceutical/nutraceutical grade l-citrulline and watermelon extract have demonstrated some efficacy in reducing blood pressure in both pre-hypertensive and hypertensive patients. Compared to placebo, treatment with watermelon extract containing 6 g/day of l-citrulline/l-arginine for 6 weeks exhibited reduced ankle and brachial systolic blood pressure (−12 ± 4 and −15 ± 3 mmHg, respectively), ankle and brachial diastolic blood pressure (−8 ± 2 and −8 ± 2 mmHg, respectively), as well as reducing the carotid augmentation index in obese pre- and hypertensive men [86]. Short-term treatment for 7–14 days with l-citrulline (5.6 g/day) reduced arterial stiffness in healthy and overweight middle-aged men [87,88]. Obese post-menopausal women with hypertension treated with 6 weeks of watermelon extract (6 g/day l-citrulline) showed evidence of reduced arterial stiffness and aortic systolic blood pressure, as indicated by a reduction in pressure pulse wave reflection [89]. In a group of middle-aged, pre-hypertensive, men and women, 6 weeks of watermelon extract supplementation (2.7 g/1.3 g l-citrulline/l-arginine) improved peripheral vascular tone (decrease augmentation index and pulse wave velocity), and lead to a significant reduction in aortic systolic blood pressure (−9 ± 3 vs. −2 ± 3 mmHg, *p* = 0.01), and non-significant reductions in brachial artery systolic blood pressure (−9 ± 7 vs. −3 ± 7 mmHg, *p* = 0.10) compared to placebo controls [90]. l-citrulline has also been shown to promote favorable adaptations in blood vessel wall stiffness measured by pulse wave velocity and hypertensive responses to cold [86,87,88]. Reduced arterial stiffness and aortic systolic blood pressure was observed in obese post-menopausal women with hypertension after 6-week supplementation with watermelon extract (6 g/day citrulline) [89]. Collectively, the current evidence supports l-citrulline and watermelon extract as viable nutritional supplements to improve resting aortic hemodynamics in individuals with prehypertension and hypertension. Although these studies demonstrate the potential for l-citrulline and watermelon extract to improve resting blood pressure and arterial stiffness, the patient population, dose, and duration of treatment appear to impact the magnitude of these effects and warrant further investigation. Table 1 provides a list of studies examining the effectiveness of l-citrulline (via watermelon extract) on reducing blood pressure in normotensive, pre-hypertensive, and hypertensive men and women.

### 5.2. Blood Pressure Reactivity

l-citrulline appears to promote adaptations to physiological and environmental stressors to reduce vessel wall stiffness and allow for improved blood flow responses. Indeed, recent data suggest that chronic supplementation of l-citrulline reduces sympathetic activity, while increasing parasympathetic tone in post-menopausal women [93,96]. Exposure to cold induces sympathetic nervous system excitation and vasoconstriction leading to increased blood pressure [98,99]. In healthy young men, 4 weeks of l-citrulline supplementation attenuated sympathetic-mediated vasoconstriction from local (foot) cold exposure for 2 min [91]. Furthermore, two-weeks of l-citrulline treatment ameliorated the hypertensive response to whole-body cold exposure and reduced myocardial oxygen demand in healthy volunteers [88]. In overweight men, 14 days of l-citrulline did not alter resting values but did significantly improve pulse wave velocity, Augmentation Index (Aix), and aortic systolic and diastolic blood pressure response to isometric handgrip exercise and cold exposure (alone and combined) [94]. Overall, these studies suggest that l-citrulline is beneficial in reducing post-exercise or cold exposure hypertension but, less consistent effects are observed for central arteries under resting conditions.

### 5.3. Long-Term Blood Pressure Regulation and Kidney Function

With respect to long-term blood pressure regulation, renal function plays a critical role, with renal vascular constrictor-dilator balance contributing significantly. In fact, all inheritable forms of hypertension involve some change in renal sodium balance [100]. Moreover, recent literature suggests that fetal programming in the kidney controls blood pressure regulation in adulthood [101], and numerous pre-clinical studies now suggest that maternal supplementation with l-citrulline can have a positive long-term impact on blood pressure regulation in offspring. For example, maternal supplementation of spontaneously hypertensive rats with l-citrulline attenuated NO deficits in the kidneys of the offspring and prevented their development of hypertension [102]. Because maternal diabetes contributes to complications in pregnancy and may impact adult diseases in the offspring, researchers also tested whether maternal diabetes induced by streptozotocin might induce renal disease and hypertension in offspring once they reach adulthood. The offspring indeed exhibited hypertension, kidney injury, and reduced levels of renal NO coupled to increased levels of ADMA. On the other hand, perinatal l-citrulline supplementation prevented the development of hypertension and the imbalance in ADMA and NO levels. Similarly, maternal supplementation with l-citrulline prevented hypertension and ADMA/NO imbalance in rats treated prenatally with dexamethasone [103]. Despite all these positive findings for perinatal enrichment, supplementation of rats during the pre-weaning stage was shown to predispose the animals in adulthood to lipid profiles that could potentially culminate in liver disease [104]. Thus, additional studies will be necessary to ensure the safety of l-citrulline supplementation at all stages of life.

### 5.4. Cardiac Function

Although both acute and chronic blood pressure regulation can profoundly affect cardiac function, few studies have systematically examined the impact of citrulline supplementation on cardiac function as attested by two recent reviews [14,21]. Indeed, acute elevations in pulmonary and peripheral vascular resistance can directly reduce stroke volumes and concomitantly reduce cardiac outputs produced by right and left ventricles, respectively. However, to ensure sufficient delivery of blood to meet the demands for both pulmonary and peripheral gas and nutrient exchange, mean arterial pressure (MAP) increases at the expense of maintaining the appropriate cardiac output to meet the prevailing metabolic demands. For example, age-related elevations in arterial stiffness [105] and vasoconstrictor tone [106] directly contribute to elevations in total peripheral resistance and lead to elevations in MAP. Chronic elevations in MAP are associated with the development of heart failure with preserved ejection fraction (HFpEF) [107], especially in older obese females. Moreover, pulmonary hypertension is also commonly observed in patients with HFpEF [107]. Of interest, l-citrulline supplementation in patients with HFpEF has been shown to improve right ventricular function by increasing right ventricular ejection fraction (stroke volume/end diastolic volume) that has been attributed to decreases in systolic pulmonary artery pressure [92]. In another study, l-citrulline supplementation increased left ventricular ejection fraction in patients with heart failure with reduced ejection fraction (HFrEF) [108]. In this study, improvements in endothelial function are supported by elevations in maximum amplitude time/total time. Collectively, these two studies suggest that l-citrulline supplementation may have beneficial effects on cardiac function, especially in patients with advanced diseases related to reduced nitric oxide bioavailability and increased vascular resistance. Future studies are also warranted to examine whether chronic l-citrulline supplementation can prevent or slow the conversion rate of hypertensive patients to these debilitating diseases.

## 6. Protection against Diabetic Vascular Dysfunction

The pathogenesis of T2D involves numerous complications of both the micro- and macrovasculature. Vascular disease is the primary cause of morbidity and mortality in T2D [109,110]. To that end, the reduction of systolic blood pressure is a focus of treatments to reduce the risk of cardiovascular events and nephropathies in patients with T2D [111,112]. The pathogenesis of vascular disease originates primarily from endothelial dysfunction [113,114,115]. Endothelial dysfunction in patients with T2D is likely due to multiple factors including chronic hyperglycemia, ADMA, ROS [116,117,118], and advanced glycation end-products [119,120,121].

Functionally, the reaction of NOS catalyzes the conversion of l-arginine to NO and the formation of l-citrulline (Figure 2). As indicated in Figure 1, circulating l-citrulline is converted to l-arginine in the proximal tubules of the kidney. This pathway is the primary mechanism for endogenous l-arginine production. However, chronically elevated glucose levels can inactive the NOS cofactors, in the kidney and endothelium, leading to uncoupling of NOS and therefore reducing citrulline concentrations [121,122].

## 7. Skeletal Muscle Heath

### 7.1. Protection against Insulin Resistance

In the case of prediabetes and overt T2D there is profound insulin resistance in the liver, skeletal muscle, and adipose tissue [123,124]. The interaction of insulin with its receptors on the plasma membrane of target tissues and the impairment of the signaling cascade can result in the inability to translocate GLUT4 to the plasma membrane, which is especially true in skeletal muscle. This manifests in decreased glucose disposal and hyperglycemia. Skeletal muscle insulin resistance has been a focus in this field due to the large percentage of glucose clearance by the skeletal muscle (>80%) during hyper-insulinemic states [125]. However, kinetic studies have demonstrated that blood flow to skeletal muscle, mediated by the vasodilatory actions of insulin, may account for differences in insulin action on glucose clearance [126,127]. As stated previously, impairments in NO signaling have both direct and indirect effects on skeletal muscle metabolism that likely contribute to the development of insulin resistance and type 2 diabetes [25,26]. In young healthy people, the vasodilatory role of postprandial hyperinsulinemia may increase vessel diameter to the extent that additional l-citrulline may not promote further improvement [126]. However, in subjects with impaired insulin secretion, particularly those with limitations in early-phase insulin secretion, supplemental l-citrulline could potentially serve to increase arginine-NO-mediated vasodilation [126].

### 7.2. Protection against Muscle Protein Loss/Wasting

The maintenance of skeletal muscle health throughout the lifespan has received considerable attention over the last few decades, due not only to its importance in maintaining cardiometabolic health (e.g., insulin sensitivity) as noted above but also in its essential role in maintaining functional independence as we age [128]. Indeed, losses of skeletal muscle mass, strength, and power are hallmark clinical manifestations of (sedentary) aging often referred to as sacropenia and/or dynapenia of aging [129,130,131]. Likewise, (sedentary) aging is also associated with declines in mitochondrial oxidative capacity and concomitant reductions in submaximal and maximal exercise performance [132,133,134]. Although age-related decreases in physical activity clearly exacerbate the clinical manifestations of sarcopenia, dynapenia, and related disorders, age-related impairments in the anabolic response to feeding (i.e., “anabolic resistance”) [135] also likely contribute to these clinical manifestations.

Supplementation with essential amino acids, especially the branched chain amino acids (BCAAs), to improve skeletal muscle and cardiometabolic health has received considerable attention over the last three decades [136,137,138,139,140]. This is particularly true for leucine, due to its well-understood role in stimulating muscle protein synthesis (MPS) [129,141,142]. Mechanistically, leucine stimulates MPS by activating the mammalian target of rapamycin complex 1 (mTORC1), which in turn increases skeletal muscle protein translation [143,144,145]. Moreover, leucine supplementation combined with carbohydrate feeding also effectively enhances acute resistance exercise induced MPS in young adults [143]. However, as stated above, recent data suggest that older adults develop anabolic resistance to mixed meal (essential amino acids and glucose) feeding leading to lower postprandial MPS in older adults compared to younger adults [135]. In a follow-up study, the same authors used contrast enhanced ultrasound to demonstrate that the age-related reductions in insulin-mediated MPS was associated with reductions in nutritive blood flow [25]. Moreover, pharmacologically inducing vasodilation restored insulin-mediated MPS in older adults to similar levels seen in young adults [25]. One could posit that supplemental l-citrulline could help restore postprandial MPS in older adults by improving skeletal muscle blood flow as a result of enhancing NO bioavailability in endothelial cells as well as potentially having direct effects on MPS.

Oral l-citrulline has recently been shown to increase MPS in healthy adults on a low protein diet [39]. However, as stated above, MPS is stimulated by both direct and indirect activation of mTORC1 [143,146]. Hyperaminoacidemia is considered a metabolic trigger to activate mTORC1 and increase MPS [146,147]. This flood of amino acids, as with other nutrients, is largely dependent on blood flow to the skeletal muscle that is often impaired with aging and in individuals with cardiometabolic diseases [25,148]. However, studies in cultured C2C12 myocytes showed that l-citrulline is capable of directly protecting against muscle wasting by preserving MPS [149]. Mechanistic studies indicate that l-citrulline exerts its effects on MPS in an iNOS dependent, but mTORC1 independent manner [149]. Collectively, the activation of these pathways likely requires other circulating essential amino acids and suggest that citrulline works by increasing the delivery of direct modulators of MPS.

Studies in aged malnourished rats have shown that l-citrulline can promote MPS during periods of malnourishment or low protein intake, in part by inducing higher insulin secretion [150]. A human trial tested the hypothesis that elderly participants with anabolic resistance to increased protein intake would experience elevations in MPS following resistance training with the co-ingestion of l-citrulline and whey protein [44]. Supplemental l-citrulline increased l-arginine availability, but in contrast to the rodent studies, there were no improvements in femoral artery blood flow or MPS [44]. These results are consistent with other studies demonstrating the biochemical increase in l-arginine without a functional improvement in blood flow, indicating l-citrulline alone may not sufficiently stimulate NO-mediated vasodilation to increase MPS [12,24]. Another recent randomized controlled trial, in malnourished older adults, also suggests that 10 g of l-citrulline per day for 3 weeks produced similar levels of whole-body protein synthesis compared to those receiving isonitrogenous non-essential amino acid supplementation [151]. However, in a subgroup of women, the supplementation with l-citrulline for three weeks did increase lean body mass [151]. We posit that older women may potentially benefit more from the vasodilatory effects of l-citrulline on MPS thus accruing more muscle mass, due to their tendency to have greater age-related reductions in skeletal muscle vasodilatory capacity and blood flow [152,153].

### 7.3. Enhancement of Mitochondrial Oxidative Capacity

Several studies have shown that the generation of NO by NOS can increase mitochondrial biogenesis (e.g., mitochondrial protein synthesis) by activating PGC1-α [154,155,156]. Nisoli et al. [154] were the first to report that NO generated by eNOS can drive mitochondrial biogenesis in multiple cell types. Moreover, treating L6 myotubes (muscle cells) with NO donors, S-nitroso-N-penicillamine or diethylenetriamine-NONO, led to activation of AMPKα1 and increased expression of PGC1α and concomitant elevations in basal respiration and maximal mitochondrial oxidative capacity [157]. Mechanistically, high levels of NO can also directly inhibit the mitochondrial transport system [156], which in turn may induce energy stress within the muscle cells leading to elevations in AMPKα1 activity that stimulate PGC1-α and mitochondrial biogenesis. Since skeletal muscle cells express both eNOS and nNOS, both isoforms could be potential therapeutic targets to mediate NO-induced mitochondrial biogenesis [156]. Of interest, recruitment of nNOS to the nucleus has been shown to be an important step in NO-induced mitochondrial biogenesis [158]. In a recent study, supplementing l-citrulline (250 mg/kg) for 15 days in mice resulted in elevations in PGC1-α mRNA and protein expression in hindlimb and forelimb muscles [159]. Importantly, these elevations in PGC1-α mRNA and protein expression were associated with improvements in exercise performance as measured by time to exhaustion during a standardized swim test [159]. Although some pre-clinical studies support the potential therapeutic benefit of l-citrulline supplementation on skeletal muscle bioenergetics, future clinical studies to determine the potential therapeutic benefits of l-citrulline in older adults and/or humans with mitochondrial disorders are warranted.

## 8. Adipose Tissue and Lipolysis Effects of l-citrulline

The l-Arginine-NO pathway is highly involved in the breakdown and oxidation of fatty acids in adipose tissue, as well as differentiation of adipocytes [160]. Obesity is associated with increased expression of iNOS, which may increase NO synthesis and contribute to metabolic dysregulation in adipocytes [161]. iNOS appears to be a critical factor in regulating cytokine (tumor necrosis factor-alpha/TNF) induced lipolysis that occurs during chronic inflammation [162]. Interestingly, selective iNOS inhibition increases TNF induced lipolysis [162]. A more global investigation of NO-mediated lipolysis demonstrates that specific NO donors (nitrosothiols) can increase basal lipolysis [163] but inhibit isoproterenol (beta-adrenergic) induced lipolysis [164,165]. These studies suggest there is a regulatory loop related to the availability of NO and its role in basal or beta-adrenergic stimulated lipolysis.

l-arginine supplementation has been shown to increase NO synthesis as well as isoproterenol stimulated lipolysis and fatty acid oxidation in the adipose tissue of ZDF rats [166]. To date, only a few experiments have examined the impact of l-citrulline on lipolysis [167,168,169]. One study has demonstrated that a 24 h treatment of l-citrulline (2.5 mmol/L) was effective at increasing basal lipolysis, reducing glyceroneogenesis, and increasing palmitate oxidation (^14^C-palmitate) in white adipose tissue explants [168]. The authors of this study reported increased expression of macrophage markers (f4/80 and CD68) and inflammatory cytokines (IL-6 and TNF) in the high fat diet (HFD) condition. It is well established that the chronic inflammation present during diet-induced obesity is characterized by the infiltration and alteration of macrophage populations in adipose tissue. Therefore, the lipolytic effects of l-citrulline reported ex vivo could be mediated by the NO produced by adipose tissue macrophages and not the adipocytes.

Treatment of adipose explants with l-citrulline has also been shown to increase the expression of UCP1, PPAR and PGC1-α in young (2–4 months), but not old (25 months) rats [167]. The observation that l-citrulline increases lipolysis and fatty acid oxidation while reducing re-esterification is somewhat unexpected. During lipolysis, there is a change in cellular energy demand that results in altered AMPK phosphorylation. In fact, AMPK levels are induced during lipolysis; however, this response is largely due to acylation of fatty acids (i.e. re-esterification) [170]. Therefore, it would be counterintuitive that l-citrulline would reduce re-esterification and increase energy consumption (fatty acid oxidation). A more plausible explanation for increased energy consumption observed with l-citrulline treatment is the increased mitochondrial uncoupling that has been reported [167]. A cell autonomous role for l-citrulline in the adipocyte has yet to be explored.

## 9. Summary and Future Directions

Increasing numbers of studies now suggest that pharmaceutical/nutraceutical grade l-citrulline and watermelon extract supplementation can increase the bioavailability of l-arginine and subsequently lead to elevations in NO synthesis. Moreover, accumulating data suggest that short-term l-citrulline supplementation can reduce peripheral and central (aortic) blood pressures in pre- and hypertensive adults. There is also evidence that l-citrulline supplementation, when compared to l-arginine, attenuates blood pressure reactivity to acute sympathetic stimulation (e.g., cold pressor and isometric/intermittent handgrip exercise) and pre-clinical (animal) evidence that it may protect against acute endothelial dysfunction. The improvement in these indicators of vascular health and resilience, and the lack of adverse side effects make l-citrulline an attractive non-pharmaceutical agent for populations at heightened cardiometabolic risk. From a metabolic standpoint, l-citrulline has been shown to increase mitochondrial biogenesis and MPS in skeletal muscle, while pre-clinical data suggests that adipose tissue lipolysis is improved with l-citrulline. Thus, l-citrulline supplementation represents an attractive non-pharmacological approach for increasing NO bioavailability, which may have the potential to counteract many of the age- and/or lifestyle-related diseases currently plaguing our society.

A major limitation of l-citrulline research to date is the short-term nature of most of the intervention periods of study. Long-term studies (>6 months) investigating the effects of l-arginine supplementation have demonstrated a concerning lack of safety and efficacy with respect to improvements in blood pressure regulation [171,172]. Basic mechanistic studies in human umbilical endothelial cells have shown that repeated exposure to l-arginine promotes oxidative stress by increased superoxide formation and reduced eNOS protein expression [173]. It is thus plausible that prolonged exposure to l-arginine promotes cellular tolerance and maladaptation. The safety and efficacy of long-term l-citrulline supplementation therefore requires further investigation. Finally, except for pharmacokinetic studies, most l-citrulline supplementation studies fail to report the plasma/serum concentrations that were achieved.

In conclusion, l-citrulline is a unique amino acid that exerts its effects on cardiometabolic health via direct and indirect pathways. The variety of applications for which l-citrulline has been utilized underscores the importance of l-citrulline in vascular health, protein metabolism, and lipid metabolism. However, the direct role of l-citrulline, outside of its function as a precursor of l-arginine, is still not well characterized. The interaction of l-citrulline with other pharmaceutical drugs for the treatment of hypertension, atherosclerosis, insulin resistance, T2D, and cardiovascular disease should also be investigated, as some of these drugs have been shown to affect citrulline metabolism [13,174,175].

## Figures and Tables

**Table 1:** A series of human clinical trials that investigated changes, after l-citrulline or watermelon extract supplementation, in blood pressure and associated indices of blood vessel function under resting and physiologically stressful conditions.

Reference	Population			BP Status	Formulation	Dose	Duration	Resting Function	Results
									Cardiovascular Reactivity
Figueroa et al. (2010) [91]	17 M			Normotensive	l-Citrulline	6 g/day	4 weeks		↓ bSBP, aSBP, aPP
Orozco-Gutierrez et al. (2010) [92]			9 M 6 F	Heart failure w/ preserved EF	l-Citrulline- Malate	3 g/day	8 weeks	↓bSPB, bDBP,	↑ RVEF during exercise
Figueroa et al. (2011) [90]		4 M 5 W		Pre-hypertensive	Watermelon Extract	2.7 g/day	6 weeks	↓bPP, aSBP, aPP, AIx	
Figueroa et al. (2012) [86]		3 M 11 W		Pre-hypertensive	Watermelon Extract	2.7 g/day	6 weeks	↓ ankle SBP, DBP, MAP, ↓ bSBP, bDBP, bMAP ↓ carotid AIx	
Figueroa et al. (2013) [89]		12 W		Hypertensive Post-menopausal	Watermelon Extract	6 g/day	6 weeks	↓b-aPWV, aSBP, aDBP, aSBP2 ↔ AIx	
Sanchez-Gonzalez et al. (2013) [88]	16 M			Normotensive	l-Citrulline	100 mg/kg	2 weeks		↓ CI and IHG increases in bSBP, aSBP and AIx
Alsop et al. (2016) [93]	4 M 8 F			Normotensive	l-Citrulline	3 d/day	1 week	↓ bSBP, bDBP, MAP, pulse interval	↓ Pulse interval, Pulse Amplitude Ratio, ↑ HRV post 30% MVC exercise
Figueroa et al. (2016) [94]	16 M			Normotensive Overweight/obese	l-Citrulline	6 d/day	2 weeks	Attenuated the increase in aSBP and AIx during IHG and reduced MAP aDBP	↓ aSBP, aPP, AIx during IHG ↓ aDBP, MAP, AIx during PEMI ↓ aSBP, DBP, aPP, an baPWV during PEMI + CPT
Bailey et al. (2016) [81]	8 M			Normotensive	Watermelon Juice	~3.4 g/day	2 weeks	↑ aSBP and MAP	
Massa et al. (2016) [95]		10 M 10 W		Pre-hypertensive	Watermelon Extract	6 g/day	6 weeks	↓ bSBP and bDBP ↔ cardiac autonomic function	
Wong et al. (2016) [96]			25 F *	Normotensive/ Pre-hypertensive	l-Citrulline	6 g/day	8 weeks	↓bSBP, bDBP, and nLF (SNS activity), LnLF/LnHF (sympathovagal balance)	
Gonzales et al. (2017) [97]			12M 13W	Normotensive/ Pre-hypertensive	l-Citrulline	6 g/day	2 weeks	↓ seated bSBP	↑muscle blood flow during submaximal exercise in men

Abbreviations: M: male, F: female, bSBP: brachial systolic blood pressure, aSBP: aortic systolic blood pressure, aPP: aortic pulse pressure, AIx: augmentation index, DBP: diastolic blood pressure, cAIx: carotid augmentation index, CI: cold induced, CPT: cold pressor test, EF: ejection fraction IHG: intermittent hand grip exercise, MAP: mean arterial pressure, aDBP: aortic diastolic blood pressure, MVC: maximal voluntary contraction, LnLF: natural log low frequency from heart rate variability test and LnHF: natural log high frequency from heart rate variability test, ↓ decrease, ↑ increase, ↔ no significant change. * Post-menopausal women 50–65 years of age.
